# Dissecting Functional Biological Interactions Using Modular RNA Nanoparticles

**DOI:** 10.3390/molecules28010228

**Published:** 2022-12-27

**Authors:** Kaitlin Klotz, Yasmine Radwan, Kausik Chakrabarti

**Affiliations:** 1 Department of Biological Sciences, University of North Carolina at Charlotte, 9201 University City Blvd., Charlotte, NC 28223, USA; 2 Nanoscale Science Program, Department of Chemistry, University of North Carolina at Charlotte, Charlotte, NC 28223, USA

**Keywords:** nucleic acid nanoparticle, RNA motif, RNA domain, SHAPE analysis

## Abstract

Nucleic acid nanoparticles (NANPs) are an exciting and innovative technology in the context of both basic and biomedical research. Made of DNA, RNA, or their chemical analogs, NANPs are programmed for carrying out specific functions within human cells. NANPs are at the forefront of preventing, detecting, and treating disease. Their nucleic acid composition lends them biocompatibility that provides their cargo with enhanced opportunity for coordinated delivery. Of course, the NANP system of targeting specific cells and tissues is not without its disadvantages. Accumulation of NANPs outside of the target tissue and the potential for off-target effects of NANP-mediated cargo delivery present challenges to research and medical professionals and these challenges must be effectively addressed to provide safe treatment to patients. Importantly, development of NANPs with regulated biological activities and immunorecognition becomes a promising route for developing versatile nucleic acid therapeutics. In a basic research context, NANPs can assist investigators in fine-tuning the structure-function relationship of final formulations and in this review, we explore the practical applications of NANPs in laboratory and clinical settings and discuss how we can use established nucleic acid research techniques to design effective NANPs.

## 1. Introduction

Nucleic Acid Nanoparticles (NANPs) are a subtype of therapeutic nucleic acids (TNAs) exclusively composed of specialized oligonucleotides designed to carry out defined architectures and functions such as delivery of therapeutic agents, biosensing, and immunostimulation [1]. NANPs can be specified by the designer to deliver functional groups capable of modulating their biological activities while adding regulatory control to the intended function of the NANP [2,3]. NANPs can be engineered to associate with specific targets which make them useful in diagnostics as well as the targeted delivery of therapeutic agents to detect and combat disease with fewer off-target impacts than experienced with traditional delivery systems [4]. For that, the sequences amenable to interactions with receptors on cellular surfaces to facilitate NANP uptake through receptor-mediated endocytosis [5]. A 2015 study by Narayan and colleagues established that class A scavenger receptors have enhanced affinity for spherical nucleic acid nanoparticles conjugates exhibiting a high guanine content [6]. High guanine content in the oligonucleotides of the conjugates facilitated adoption of a secondary structure that facilitated uptake of nanoparticles carrying camptothecin by A549 (human lung adenocarcinoma) cells which resulted in the significantly diminished viability of the cancerous cells seven days after treatment with a G-rich spherical conjugates [6].

NANPs are often used as a method of getting nucleic acids past “barriers” that exist in the body. Typically, carrier-free, naked, exogenous nucleic acids introduced without any chemical modifications would meet one of two fates within the body: rapid nuclease-mediated degradation and/or renal clearance. The engineered larger NANPs or NANPs mixed with the delivery agents assemble in such a way that the cargo they are delivering is protected from degradative nuclease activity which would otherwise stop the NANPs from achieving their therapeutic purposes. Additionally, the architectural parameters (size, shape, composition, functionalization with TNAs, etc.) of NANPs can be engineered in a way that is non-immunogenic and allows the payload to be shuttled to its intended target without triggering any immunological responses [7]. Once the problems of nuclease mediated degradation and immunogenicity are dealt with, NANPs can deliver gene regulatory RNA interference (RNAi) inducers to decrease the expression of overexpressed genes in pathogenic settings [4,8].

### 1.1. Nucleic Acid Nanoparticle Design and Functionalization

There is more than one way to design NANPs and the method used to design a particular NANP depends on the material delivered as well as its action upon delivery [9,10,11]. The physical properties of individual NANPs determine how well the NANP interacts with its intended target and consequently, impacts the efficacy of the NANP’s payload delivery. Several computer-assisted approaches allowing for designing RNA and DNA NANPs have been introduced and explored [9,12,13,14,15,16,17]. Once a particular NANP is selected for further use, the addition of functional moieties to its structure can be achieved through a direct extension of 5′- or 3′-ends of individual strands that enter the NANP’s composition. One way in which NANPs demonstrate specificity is through aptamers. Aptamers are single-stranded oligonucleotides that can adopt specific conformations [18]. Due to their specific conformational arrangements, aptamers can interact with cell components, namely receptors and ligands, in a level of specificity like that of antibodies to elicit a response [18,19]. Their ability to modulate pathways within cells makes aptamers an attractive candidate for therapeutics-particularly in an antagonistic indication [18].

In terms of NANP design, aptamers can be added to NANPs to enhance the specificity of NANP binding. This binding of NANPs can be to whole cells (on the cell surfaces), as well as proteins, and in some special cases, viruses [20]. NANPs provide therapeutic agents access to cells without leaving those therapeutic agents subject to degradation and less prone to triggering an immune response. The aptamers, which have undergone several rounds of Selective Evolution of Ligands by Exponential Enrichment (SELEX), are the key to getting the therapeutic agent to exactly where it is needed [19,20]. We can think of aptamers like a ZIP code on a letter such that the contents of the NANP could go to several different places, but when the aptamer is added, the potential delivery locations are narrowed down to a specific place within the cell. 

### 1.2. Selection of Nucleic Acid for NANPs

In the previous section, it was stated that NANP design is influenced by the cargo that is delivered as well as the intended action. The same dependence on cargo and action must be considered when selecting the nucleic acid for a NANP. Both DNA and RNA NANPs have been developed for therapeutic and research applications, but the two have several distinct qualities that make them unique from one another. In general, DNA is typically regarded as the more stable of the nucleic acids, however, that does not mean that RNA is the “lesser” nucleic acid. In fact, RNA has some properties that make it favorable for use in NANPs. 

A clear distinction between DNA and RNA nanoparticles is the base pairing capabilities of each nucleic acid. DNA is confined to Watson–Crick base pairing, meaning that A binds with T and C binds with G. While RNA often does form Watson–Crick base pairs, it also participates in non-canonical base pairing, such as G:U, G:A or C:A type as found in RNA structural folds [21]. These noncanonical base pairs play critical roles in RNA-folding to establish the three-dimensional structures required for diverse functions of RNA. Thus, RNA’s ability to form non-canonical base pairs allows it to adopt motifs with discrete structure and function, setting it apart from DNA [21]. The motifs present in RNA provide it with enhanced thermal stability, which can be variable in relation to base pairs in DNA, creating differentials in thermal stability within the same DNA sequence [21,22]. This stability is further reflected in the free energy of RNA-RNA helices [21]. The free energy for RNA-RNA helices is the lower that of DNA-RNA hybrid helices and DNA-DNA helices [21,23,24].

For a NANP to carry out its intended function, it must be recognized by a receptor on the exterior of a target cell and endocytosed into the cell. Upon endocytosis, the NANP is inside of an endosome that functions to degrade molecules and reuse their components [21,25,26]. The endosome is acidic to aid in the degradation of endocytosed molecules [25,26,27]. In the case of DNA, this low pH environment leads to depurination-a process in which adenine and guanine (the purines) are protonated and subsequently lost from the sequence, leaving the DNA lacking purines and susceptible to degradation [21,28,29]. On the other hand, RNA is tolerant of lower pH levels than DNA, which will facilitate successful delivery through the endosome [21]. 

In summary, RNA nanoparticles are more stable thermodynamically and to pH ranges. Additionally, they have greater structural flexibility. When considered together, these features make RNAs preferred over DNAs as NANPs. 

### 1.3. Small Interfering RNA (siRNA)

**Figure 1:**
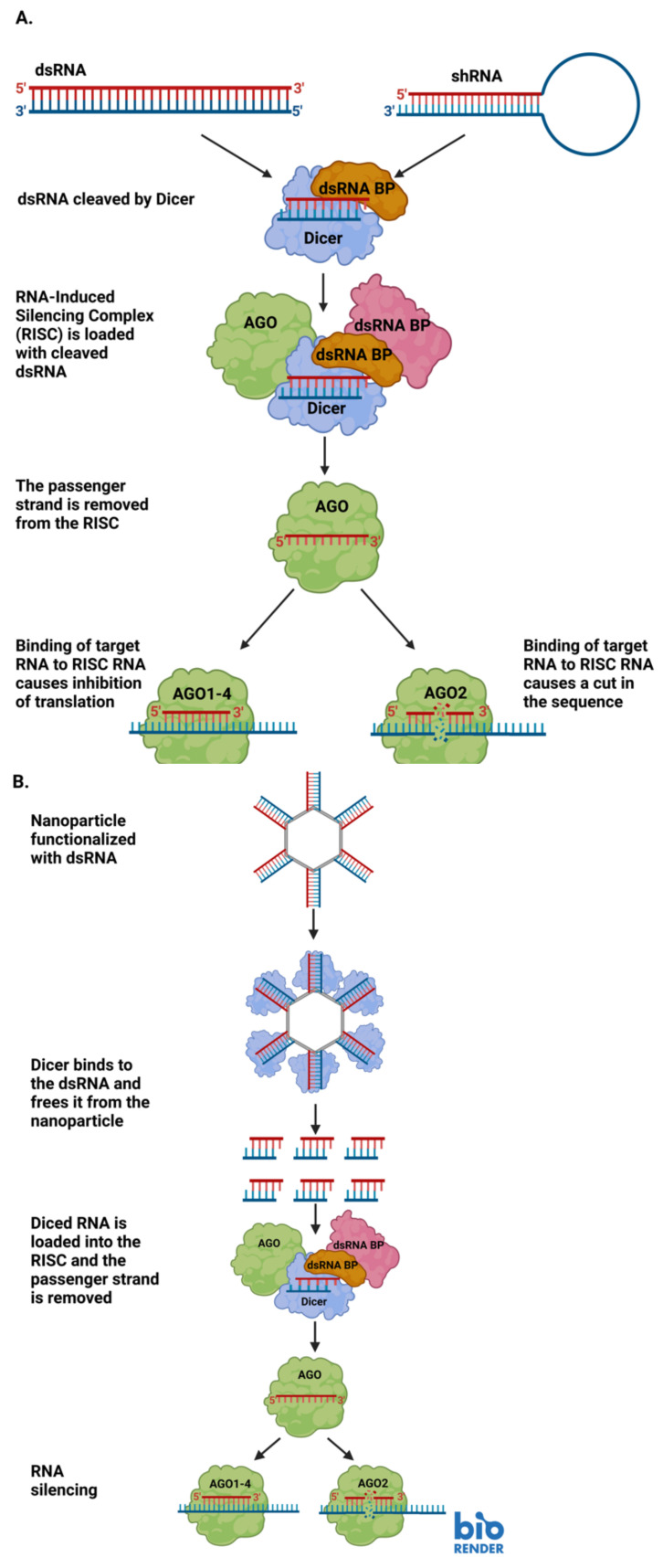
(**A**) Diagram detailing the steps involved in RNA interference (RNAi) through the production of small interfering RNAs (siRNAs). (**B**) A representative diagram of how siRNAs may be delivered to a cell via RNA-functionalized nanoparticles, and how the siRNAs can be used for RNAi upon delivery. Image created with BioRender.com.

Small interfering RNA (or siRNA) is a short, non-coding, regulatory RNA [30]. Like other small RNAs, siRNAs are generally 20–30 nucleotides in length, and they can modulate the expression of genes [30,31]. siRNAs are usually exogenous to the organisms they regulate, and they can be designed by researchers to target a specific gene for downregulation [31]. siRNAs are commonly used in RNA interference (RNAi). RNAi has long existed in nature, however, more recently researchers have begun to harness its power to regulate gene expression [32]. siRNA is used to target specific sequences of RNA or DNA. siRNA originates from double-stranded RNA (dsRNA), which is not common in cells and triggers a cascade to eliminate the dsRNA [31]. The dsRNA is cleaved into small pieces, which become the siRNAs, by a protein called Dicer [31]. siRNAs are loaded into the RNA-Induced Silencing Complex, or RISC [31]. Once in the RISC, the strands of the siRNA are separated and only one strand is retained [31]. When the siRNA-bound RISC recognizes a mRNA sequence complementary to the siRNA strand, the RISC activates a protein called slicer to cleave the mRNA and render it untranslatable [31]. Sometimes, there is not perfect sequence complementarity, but it is still similar enough that the siRNA strand will bind the partially complementary RNA and block it from efficient translation (Figure 1A) [32]. In a therapeutic setting, researchers may introduce synthetically produced siRNAs for targeted knockdown of a gene of interest. In this case, the siRNA is not cleaved from a larger dsRNA inside the cell-it is already the appropriate size for association with the RISC [31].

NANPs are particularly useful in the delivery of siRNA due to their stability [33]. Typically, “naked” RNAs are quickly recognized as foreign and degraded by endogenous nucleases inside the cell or organism to which the RNA is delivered [33]. The stability provided to the siRNA payload delivered by the NANP increases the ability of the siRNA to reach its intended target without being destroyed by the recipient. Additionally, NANPs can be conjugated to aptamers which bind with high specificity to a desired target. The specificity conferred to the siRNA delivery by nanoparticles reduces the possibility of off-target binding/delivery and increases the concentration of siRNA at the desired target [19].

### 1.4. NANP Applications in Medicine

In addition to the delivery of therapeutics to treat disease, NANPs can be used in disease prevention. RNA or DNA NANPs can be designed to deliver genetic information from pathogens in vaccines to prime the immune system for a natural exposure [34]. This is a new and exciting application of nanoparticles that we will undoubtedly see become more common as nucleic acid vaccines are further developed. NANPs can also be used as adjuvants that allow vaccine materials to be incorporated into the target tissue to stimulate an appropriate immune response [35]. On the flipside, nanoparticles can be altered to be immunologically inert and lack inflammatory activation, which can result in adverse effects to the patient. The future of NANP medicine will rely on careful optimization of these drugs and mitigation of their side effects and off-target effects.

## 2. Functionalized RNA Nanoparticles

### 2.1. RNAi

RNAi is the phenomenon of dsRNAs knocking down or silencing the expression of endogenous mRNAs initially described by Andrew Fire and Craig Mello nearly 25 years ago [36,37]. RNAi relies on small interfering RNAs (siRNAs) which silence encoded genes (Figure 1A). This occurs through a short double-stranded RNA binding with a target and preventing translation into functional protein [38]. While Fire and Mello characterized RNAi with an exogenous RNAi with dsRNA delivered from outside in C. elegans, the phenomenon is an evolutionarily conserved method of post-transcriptional gene silencing that has served as a means for organisms to protect themselves from exogenous threats as well as regulate gene expression [39]. RNAi has been characterized in plants as a sort of immune protection from plant viruses and insects [39]. A few years ago, Chejanovsky and colleagues used deep sequencing to detect the presence of perfect siRNA matches for three viruses that strongly contribute to colony collapse disorder in the genomes of honey bee colonies that had succumbed to colony collapse disorder [40]. In an experimental setting, RNAi has been used to tackle a wide range of biological problems. RNAi-mediated biological pest control has been used experimentally to protect valuable crops which will provide nutrients to countless people and livestock [41]. Building off of Chejanovsky’s work in honey bees, RNAi has been experimentally employed to combat viruses that contribute to colony collapse disorder and threaten global food supplies [42]. NANPs can be used to mediate the delivery of exogenous RNAs for RNAi within cells. NANPs functionalized with the exogenous RNA deliver the RNA inside a cell to silence a target gene and prevent the formation of that gene’s product [43]. Once the NANP has been internalized within the cell, the enzyme Dicer acts upon the attached RNAs, allowing them to participate in RNAi-mediated gene silencing (Figure 1B) [43].

Currently, RNAi is a subject of much research and development for clinical applications [44]. Despite a rocky start in the 2000s in which several RNAi therapeutic candidates were pulled from clinical trials due to unintended effects, Patisiran became the first RNAi drug to receive FDA approval in 2018 [37,45]. Patisiran treats hereditary transthyretin amyloidosis-a deadly genetic disorder which is characterized by the deposition of amyloid plaques of the protein transthyretin in key organs such as the heart and kidneys, leading to deterioration of quality of life for patients and eventual death [45,46,47,48]. Today, there are several RNAi drugs in clinical trials for treating cancers, inherited genetic disorders such as Sickle Cell Disease and familial hypercholesterolemia, and viruses such as HIV and hepatitis B [37].

Like with all new technologies, RNAi does not come without drawbacks. Naturally, we must consider immunogenicity. RNAi therapeutics work at their best when they are delivered to the appropriate tissue without degradation. When the RNAi drug triggers an immune response, the drug may never reach its intended target, or if it does, it could be in a less effective state [37]. In addition to unintended immunogenic responses, accumulation of RNAi therapeutics in unintended locations and subsequent toxicity are of major concern to RNAi drug developers [37]. While accumulation of RNAi therapeutics in off-target tissue is a very legitimate problem in and of itself, an additional unintended consequence of RNAi drug treatment is the drug acting as it is supposed to outside of its target tissue and causing interruption to normal tissue function [37].

To continue to develop the applications of RNAi therapeutics, researchers have gotten creative with how they have approached drawbacks. Researchers have incorporated base modifications into the RNA delivery system to get around the problem of immunogenic activation caused by RNAi therapeutics [5,37,49]. In 2007, Robbins and colleagues demonstrated that the addition of a 2′-O-Me modification to siRNA reduced the siRNA’s immunogenic potential without diminishing its ability to decrease expression of a target gene [49]. The added benefit of the base modifications is increased RNA stability, which generally increases the amount of time it is able to remain in the body without becoming degraded or neutralized by the immune system [5,37]. When the circulation time is increased, the RNAi therapeutic has an increased probability of arriving at its target and carrying out its intended function.

To mitigate off-target RNAi activity, researchers propose some common-sense measures to enhance safety for RNAi therapeutic recipients. The first proposed measure occurs long before the RNAi therapeutic makes it to the patient-it is extensive quality control to ensure that the drug has as few targets in the human genome as possible [37,50]. In their 2019 review, Setten and colleagues uphold that some off-target binding is inevitable and patients receiving RNAi therapy should be administered the smallest dose capable of achieving the desired effect, while being closely monitored for signs of off-target activity that is threatening to the patient’s wellbeing [37].

It becomes more challenging when on-target effects in off-target tissues are considered. The drug is doing what it is supposed to do, just in a suboptimal location, which can imperil the patient’s health. Much like mitigating off-target effects in off-target tissue, researchers must design intentional, highly specific therapeutics to ensure that there are as few possible routes for the therapeutic to build up in a non-target location [37]. In addition to designing therapeutics with the awareness of these effects, drug developers have “reverse engineered” compounds to reverse the impacts of the siRNA therapeutics in the event that they excel at performing their intended functions outside of the optimal location [37,51].

### 2.2. NANP-Induced Immunogenicity

**Figure 2:**
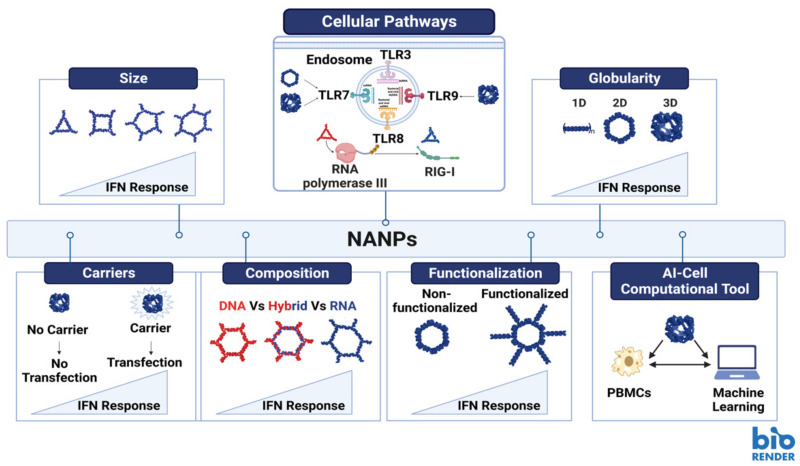
Structural parameters and other factors affecting immune stimulation by NANPs. Image created with BioRender.com.

The use of NANPs in humans as well as other animals carries the potential for immune activation. The immunogenic response must be thoroughly evaluated by the investigators to ensure that it is not activated in an unintended manner (i.e., prior to the delivery of the NANP to the target tissue). Researchers do have some (though not complete) control in how immunostimulation proceeds through the design of their NANPs. A 2017 research article by Guo and colleagues examined the impact of NANP sequence as well as physical properties (size and shape) on their immunostimulatory effects [52]. In this study, the research team demonstrated that increases in the size of RNA squares resulted in enhancement of cytokine secretion, namely TNF-α and IL-6 [52]. Similarly, RNA squares with attached uniform RNA sequences showed the same effect-as the number of attached RNA sequences increased, so did the levels of cytokine secretion [52]. The same study indicated that three-dimensional RNA nanoparticles elicited higher levels of cytokine secretion than their planar counterparts [52]. Figure 2 provides a visual representation of the results that were described in Guo’s 2017 article (Figure 2).

### 2.3. Immuno-Adjuvant

The use of adjuvants offers a robust method where immunostimulatory compounds are employed to potentiate and modulate the immune response, when used together with vaccines. Among the recent advancements of the immunomodulatory NANPs, is their ability to regulate and modulate the immune responses when encountered. The rapid clinical development of this technology is impeded by several hurdles among which is the unknown immune stimulation by NANPs. However, recent studies have reported that mammalian cells use the same patterns of recognition established to defend themselves against viral and bacterial nucleic acids to process NANPs [2]. Further investigations showed that various interactions of NANPs with different immune cells elicit different immune responses. The regulation of the immune response by NANPs can be controlled by varying their design, specifically by altering numerous architectural parameters including the NANPs’ size, shape, functionalization and composition (Figure 2) [2,8,53,54]. The correlation between those variations in NANPs’ structures and their effect on the immune response is now being considered carefully during the design process aiding to develop NANPs that would either modulate the immune activation or stay immunoquiescent [54]. Other aspects considered to successfully translate NANP technologies into clinical settings would include the choice of delivery carriers and administration routes for NANP formulations. If NANPs complexed with a carrier are delivered via intravenous administration, they may induce undesired inflammation due to cytokine induction and complement activation. However, if the same system is administered locally, it would serve perfectly as immune-adjuvant as it will induce the same cytokine and interferon response along with complement activation, which would potentiate the vaccine efficacy and enhance immunotherapy efficacy [53].

### 2.4. NANPs with Regulated Immune Responses

Human cells have receptors for the recognition of foreign nucleic acids called pattern recognition receptors (PRRs), which can also distinguish and process NANPs. Therefore, understanding the underlying mechanisms of recognition and the NANPs structural parameters that affect their recognition process would allow the tunability of the immunostimulatory effects. This, in turn, will allow engineering the immunoquiescent NANPs intended for drug delivery, or NANPs with regulated immunological properties that could be used in immunotherapies [3]. In the case of immune-adjuvants, PRR agonists help to stimulate the innate cytokine and interferon production, which endorses the cellular antiviral defenses [3].

Extensive studies have been carried out to assess the immunostimulation of the representative library of NANPs introduced to freshly collected human peripheral blood mononuclear cells (PBMCs) [3,7,8,55,56,57,58,59,60,61]. PBMCs were chosen as a highly reliable model for prediction of cytokine storm toxicity in humans [57]. One of the parameters that affects the immunorecognition of NANPs is their chemical composition (e.g., DNAs vs. RNAs vs. DNA/RNA hybrids) (Figure 2-Composition) [55,62]. A study reported that altering the composition of NANPs can modulate the mechanisms and degree of elicited immune responses [63]. It also highlighted that NANPs made of RNA normally demonstrate significantly higher immune activations in comparison to their DNA counterpart, since RNA NANPs can trigger both TLR7 and RIG-I mediated cytokine and interferon response (Figure 2-Composition) [63,64,65]. Another parameter is NANP size; increases in the size of NANPs may lead to elevated immunostimulation (Figure 2-Size). One more parameter that plays a major role in immunostimulation is the dimensionality of NANPs. Several studies have confirmed that for RNA NANPs, fibrous structures (1D) demonstrate reduced immunostimulation when compared to planar NANPs (2D) and that the globular RNA NANPs (3D) produce the highest levels of immune responses amongst all of them (Figure 2-Globularity) [2,59,60]. It was reported that 2D and 3D RNA NANPs induced interferon production upon activation of TLR7, while 1D NANPs did not [62]. In addition, it was shown that the immunostimulation of NANPs functionalized with therapeutic nucleic acids (TNAs) induce higher production of type I and II IFNs when compared to non-functionalized NANPs and that the extent of activation can be regulated by relative orientation of the TNAs (Figure 2-Functionalization) [8,60].

### 2.5. The Role of Carriers on NANPs Immunorecognition

Another hurdle precluding broader clinical application of NANPs is their intracellular delivery [1,66,67]. One of the most essential factors for NANPs delivery and their efficacy is the use of carriers and complexation agents. Extensive studies had previously reported that carrier-free NANPs do not elicit any immune response as they are invisible to the cells, and the use of delivery platforms further tailors the immunorecognition of NANPs [2,53,68]. Hence, for efficient intracellular delivery of NANPs, various delivery agents such as lipid-based carriers [55], exosomes [69], polymeric agents [70], and inorganic materials [71,72] have been investigated. One study employed PBMCs to investigate the use of amine-terminated PAMAM dendrimers to deliver NANPs (e.g., RNA and DNA cubes) and compared to commercially available Lipofectamine 2000 (L2K), a well-established lipid-based delivery platform [55]. The results highlighted that the uptake of the NANPs by different human immune blood cells, and their cytokine responses varied based on the delivery system used [55]. NANPs complexed with dendrimers did not induce type I and type III IFNs as opposed to NANPs complexed with L2K. In addition, NANPs complexed with L2K did not induce cytokine production (IL-1α, IL-1 β, IL-6, TNFα), while NANPs complexed with dendrimers induced the production of these stress associated cytokines. The 3D RNA NANPs (RNA cubes) delivered by dendrimers also elicited a more potent profile of cytokine production when compared to their DNA counterparts, which aligned with previous findings highlighting the effect of NANP composition on immunorecognition [53]. Additionally, as was expected, the carrier-free NANPs did not elicit any immune responses [55]. To overcome the barrier of safe and efficient delivery of NANPs while avoiding the carrier-associated toxicity, naturally occurring nanovesicles involved in cellular communication (e.g., exosomes) can be utilized. The exosomes provide a stealth-coating for loaded NANPs, which prevents nuclease degradation of NANPs as well as exposure to PRRs. For example, exosome-mediated delivery of RNA cubes which are known to have high immunostimulatory effects on cells, showed negligible immune activation [69], as compared to other carriers.

As each set of NANPs holds a unique physicochemical and architectural profile, this creates a burden to predict the type of immune response and its magnitude. To overcome this challenge, a computational predictive tool called “artificial immune cell”, or AI-cell, was developed to guide the design of NANPs to fit the desired immunological profiles. This unprecedented computational approach is fed by physicochemical and immunological profiles for an array of various NANPs and uses innovative transformer architectures to predict the immunological activity of NANPs based on the entered oligo and their sequence compositions [7]. This freely available web-based implementation is expected to advance the understanding of properties that contribute to immunomodulatory activity of NANPs and draw guidelines for their design principles. The AI-cell shall further promote the therapeutic nucleic acid nanotechnology even further by addressing the public health challenges related to the toxicities of nucleic acid therapies [7].

## 3. RNA Motifs and Domains, and Their Delivery via Nanoparticles

### 3.1. Motifs

In 1999, P.B. Moore defined an RNA motif as a “discrete sequence or combination of base juxtapositions found in naturally occurring RNAs in unexpectedly high abundance” [73]. Moore’s definition of an RNA motif leaves room for RNA sequence motifs as well as structural motifs [74]. RNA motifs occur naturally, can exhibit three-dimensional structure, and can interact with other motifs in RNAs and protein domains to contribute to their overall functionality [73]. Researchers and pharmaceutical developers have taken advantage of naturally occurring RNA motifs and incorporated them into their nanoparticle designs for enhanced stability and increased capacity for payload delivery and tracking [5].

### 3.2. Domains

In a 2002 review of protein domains, Ponting and Russell provided three different perspectives from which protein domains could be defined: biochemical, structural, and sequential [75]. Structurally, they defined domains as “spatially distinct units” [75]. In the biochemical context, they were less concerned with structure and specified a domain as a region with a clear-cut function [75]. From a sequential standpoint, Ponting and Russell claim that a domain is characterized by homology to other sequences which achieve similar functions in different environments [75]. While each definition of the domain holds some truth, all three should be considered together to get the full understanding of the domain.

Ponting and Russell did provide a more modern definition of a domain as a structure that could adopt the necessary structural conformation for carrying out its function [75]. While this definition is applied to protein domains, it could be applied in the context of RNA as well. After all, RNA can adopt specific conformations that facilitate biological functions. RNA motifs, described earlier, have the capacity to build upon each other and interact with other motifs in ways that perform biological functions. Those interactions between motifs are key to the establishment of RNA domains. A 2011 review by Reiter, Chan and Mondragón described domains as complex, functional, three-dimensional structures in the RNA that are comprised of (and stabilized by) interacting RNA motifs [76]. Functional RNA motifs are the building blocks of larger functional RNA domains that have unique three-dimensional structures [76].

### 3.3. Motifs and Domains in Research

Since the late 1980s, RNA motif research has been rapidly growing. In a 1998 review article, Conn and Draper claim that there are only a few functional RNA motifs, but when these motifs are placed together in combination, the functions that can be carried out by the structured RNA and the specificity with which these functions can be executed is enormous [77]. Nanoparticles, which were in their early days at the time of Conn and Draper’s review, take full advantage of combinatorial effects of RNA motifs. RNA motifs themselves are quite frequently incorporated into the designs of nanoparticles for their functionality [5].

RNA tectonics (or tectoRNAs) utilize naturally occurring RNA motifs to form hierarchically folded modular functional RNAs which can be used to construct RNA nanostructures [78,79] and illuminate the functions of already-existing RNA structures [80]. We can think of modular tectoRNAs as jigsaw puzzle pieces that when put together, provide us with the greater unified function of the specific nanostructure much like the pieces of an actual jigsaw puzzle show us an entire picture [79]. However, unlike a jigsaw puzzle, tectoRNAs (or pieces) can be reused in different nanostructures (puzzles) to make and execute entirely new functions [80].

### 3.4. RNA Functional Augmentation

Over the last nearly three years, the public has become increasingly aware of the use of RNA in medicine through the SARS-CoV2 vaccine. Two of the three mainstream COVID-19 vaccines contain the mRNA transcript encoding the spike protein, which facilitates viral entry into the cell [81]. The mRNA to be delivered is encased in lipid nanoparticles to prevent rapid degradation of the encoded instructions [81]. Once delivered, the mRNA becomes translated into the viral spike protein and takes on its unique conformation to elicit an immune response against the spike protein [82]. The nanoparticle delivery facilitates non-immunogenic delivery of the mRNA cargo which is selected for its ability to trigger immune activation.

Outside of the COVID-19 context, RNA nanoparticle delivery could be used for functional complementation studies. Previously, conjugates of gold nanoparticles were used as carriers for functional RNA structures in cells and these in-cell structures efficiently contribute to gene expression regulation [83]. X-ray crystallographic determination has provided further evidence that these nanostructures can fold into stable RNA motifs, such as kissing loops and T-junctions, that resemble natural RNA motifs [84]. This opens up the possibility of using RNA structural motifs as nanostructures for genetic complementation studies to restore a normal phenotype to mutants with some defect (Figure 2-Functionalization). In this arena, our team is exploring how crucial discrete structural domains of RNA can be used as nanostructures to compensate functional deficiency in parasitic disease caused by the protozoan parasite *Trypanosoma brucei*, the causative agent of African sleeping sickness. Telomerase RNA is a long noncoding RNA that is an integral functional subunit of a large RNA-protein complex responsible for synthesizing the G-rich strand of telomeres, the physical ends of linear chromosomes. Telomerase is critical for telomere length maintenance, thus preventing chromosome instability in eukaryotic cells [85,86]. Our previous and ongoing works with *T. brucei* telomerase RNA structural domain deletion mutants have demonstrated that certain domains of the telomerase RNA are vital to cell proliferation [87]. Once the functions of the *T. brucei* structural domains have been established, we aim to deliver the missing telomerase RNA structural domains to the domain-deletion mutants in an effort to restore their functions.

### 3.5. Domain Delivery and Associated Challenges

**Figure 3:**
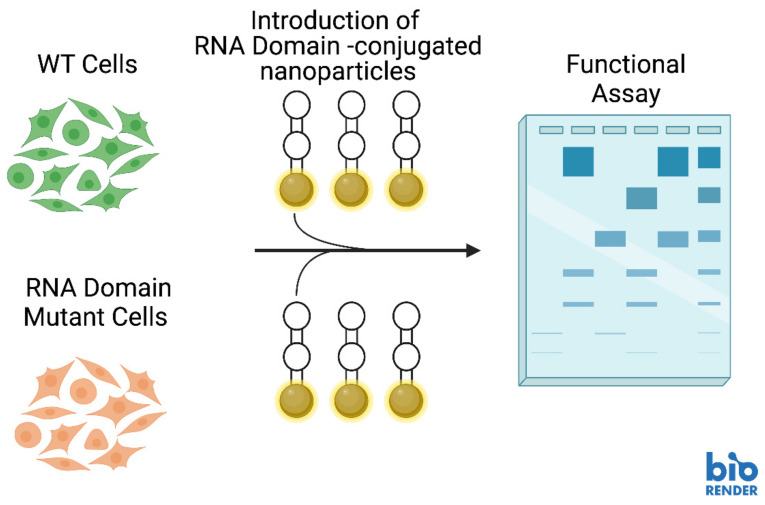
Hypothetical experimental schematic for delivering RNA structural domains to RNA domain-depleted cells for complementation studies with nucleic acid nanoparticles (NANPs). Image created with BioRender.com.

**Figure 4:**
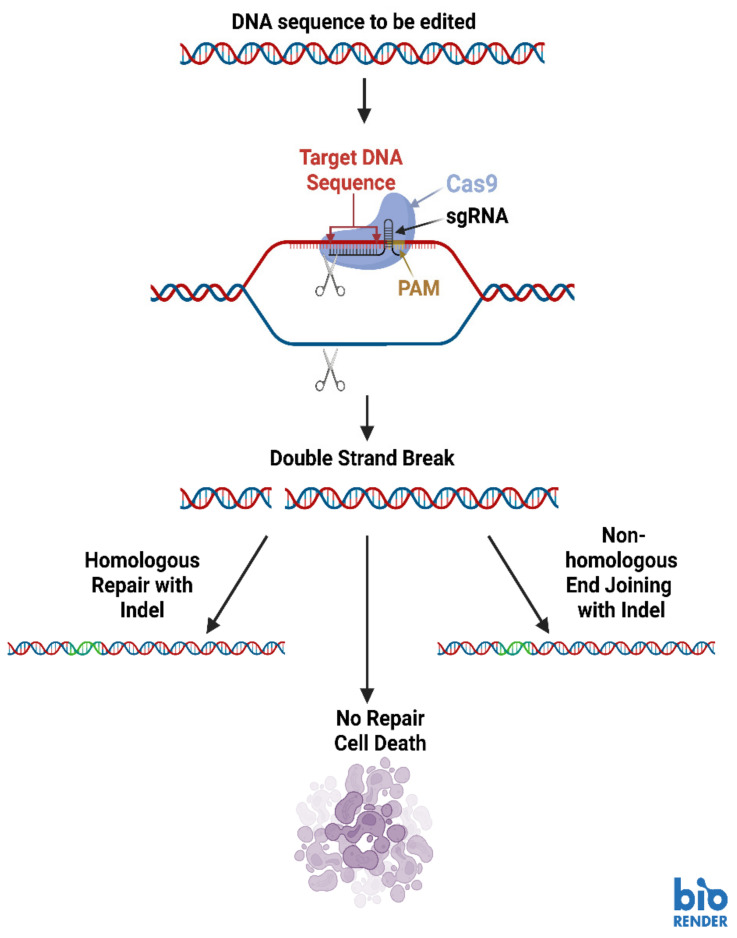
Basic diagram of the CRISPR-Cas9 genome editing system. Image created with BioRender.com.

Several different types of RNA can be delivered to cells for purposes such as post-transcriptional regulation, enhancement of catalytic activity and augmentation of gene expression [88]. NANPs functionalized with aptamers can deliver cargos (including RNA domains) to highly specific locations within cells (Figure 3) [88]. Additionally, CRISPR (Clustered Regularly Interspaced Short Palindromic Repeats) technologies are showing great promise in targeting highly specific sequences for editing (Figure 4) [88]. CRISPR genome editing is inspired by a bacterial immune system to protect bacteria against viral invaders [89]. The CRISPR-associated (Cas9) restriction enzyme is directed by a guide RNA (gRNA) which binds to a protospacer adjacent motif (PAM) on a segment of DNA containing the target for editing [89,90]. Cas9 moves from the PAM to determine if there is base complementarity between the gRNA and the DNA target sequence [89,90]. When complementarity between the gRNA and target DNA is located, the Cas9 endonuclease creates a double strand break (DSB) in the target DNA sequence [89,91].

Once the DSB has been made, the host cell’s DNA damage repair machinery is activated to prevent cell death [89,92]. Repair processes such as non-homologous end-joining (NHEJ) and homology directed repair (HDR) commonly activate and they cause alterations in the target sequence [89]. A single DSB repaired through NHEJ often incorporates extra base pairs which interrupt the coding sequence and result in the absence of the gene product (Figure 4) [89]. If two different gRNAs are used to make cuts at different places in the target sequence, a segment of the target DNA can be eliminated [89]. If a DNA template is also present in the CRISPR-Cas9 reaction involving two gRNAs, HDR will incorporate the DNA template into the target sequence [89].

Although NANPs are exceptionally promising as tools for modulating gene regulation, they come with considerable delivery and stability issues. NANPs exhibit diminished stability in mammalian serum [63]. In addition to the low stability of NANPs inside of the body, RNAs are rapidly degraded inside of cells by ribonucleases. When an unstable NANP is coupled with RNA domains that could be destroyed upon cellular entry, the prospects of the RNA domains reaching their intended target decrease dramatically. Furthermore, RNA carries a net negative charge, which makes it unlikely to achieve internalization within cells without modifications or incorporation within a carrier that is more amenable to traversing the plasma membrane [2,63]. NANPs carrying exogenous RNA are also quite effective activators of immune responses which can stimulate inflammation harmful to the patient’s wellbeing, and to the successful delivery of the NANP payload.

The challenges of NANP stability and rapid RNase-mediated degradation of RNAs delivered by NANPs are not absolute. In fact, over the last decade, significant strides have been made in mitigating NANP serum instability and exogenous RNA degradation. In a 2020 article by Johnson and colleagues, they effectively demonstrated that the composition of the NANP itself carried a significant impact on how stable the NANP was in serum [63]. Replacement of the 2′-OH group in RNA by a 2′-F increased stability for triangular NANPs that contained either an RNA or DNA center [63]. In addition to enhancing the stability of NANPs, the substitution of the 2′ hydroxyl group for a fluorine assisted in mitigating RNase-mediated degradation of RNA NANPs [63,93].

## 4. Determination of RNA Structural Properties in Nanobiotechnology

### 4.1. Importance of RNA Structure Determination

It is a strong theme throughout biology that structure is crucial in determining function. The structure of RNA is dynamic and typically reflects the RNA’s specific function [94]. Whether it is the delivery of RNA nanoparticles in cells or RNAs delivered via gold or other nanostructures, determining thermodynamically stable structures, such as three-way junction (3WJ) motifs or structural RNA domains are important for further investigations. With the advent of high-throughput sequencing based RNA probing and cell-penetrating chemical probes, it is now possible to determine structures of RNAs in vivo. In our research team’s work, we have demonstrated the dynamic nature of RNA through different stages in the life cycle of *Trypanosoma brucei* [87]. The needs of cells can change throughout their life cycles and having an understanding of how the changing structure of RNA contributes to meeting those needs is of the utmost importance [95].

### 4.2. Methods for Structural Prediction

**Figure 5:**
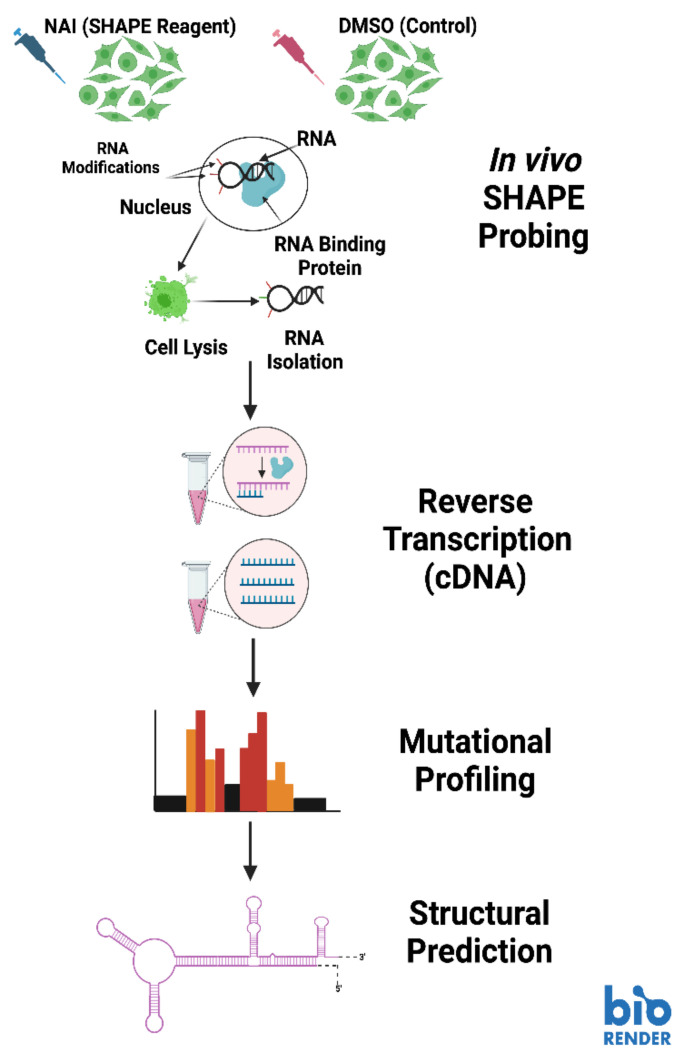
The SHAPE-MaP pipeline for RNA structural analysis. Image created with BioRender.com.

In some of our recent work, we employed a selective 2′-hydroxyl acylation analyzed by primer extension mutational profiling (SHAPE-MaP) technique to model telomerase RNA secondary structure at different stages of the life cycle in *Trypanosoma brucei* (Figure 5) [87,96]. We chose this technique for its ability to visualize RNA conformations within living cells, as well as its adaptability for immunoprecipitated RNA [87]. The SHAPE protocol works by applying a SHAPE chemical probe to a sample to facilitate the addition of bulky adducts to RNA bases that are not engaged in a binding arrangements with other bases (from the same RNA or a different RNA), protein, or DNA [96]. For the purpose of comparison, RNA extracts are also treated with a control that does not place bulky adducts on the unbound bases, which is usually the solvent for the SHAPE reagent [96]. Once the adducts are associated with the unbound bases, library preparation is started through the production of cDNA, which will induce mutations in the sequence at the location of SHAPE reagent-inflicted adducts. A second strand of DNA is made to stabilize the DNA libraries and prepare them for high-throughput DNA sequencing. The sequences from the SHAPE reagent treated samples is compared with the sequence data from the control treated samples, then aligned to identify where the SHAPE reagent induced mutations are located (Figure 5) [96]. The sequence data is processed by the SHAPE-MaP software program to calculate the flexibility (reactivity) at each base position [96]. Both the sequence data and the flexibility data are used in a structure prediction program (we used RNAstructure) to create minimum free energy models of the RNA secondary structure [87,96,97].

More recently, a new application of selective 2′-hydroxyl acylation analyzed by primer extension emerged as juxtaposed merged pairs protocol (SHAPE-JuMP) [98]. SHAPE-JuMP serves the purpose of supporting higher-order RNA structural prediction through crosslinking nearby structures in the same RNA transcript [98]. The crosslinker is a SHAPE reagent called trans-bis-isatoic anhydride (TBIA) which has two functional sites that interact with the 2′ hydroxyl groups of bases in RNA structural domains that are near to each other [98]. Once the RNA structures are crosslinked, RT-C8, a reverse transcriptase capable of “jumping” over the TBIA crosslinker, reverse transcribes the RNA into DNA while leaving out the RNA between the crosslinked bases [98]. The skipped region of RNA appears as a deletion when the sequence is aligned to a reference sequence [98].

### 4.3. Advantages and Limitations of Structural Modeling

A crucial advantage of using SHAPE-MaP techniques to predict the secondary structure of RNA is the ability to use it inside living cells in addition to outside of the cells and on deproteinized “naked” RNA [96]. In their protocol paper, SHAPE-MaP developers Smola and Weeks propose using both in-cell and cell-free SHAPE-MaP procedures to identify the locations of likely RNA-protein interactions [96]. Since the SHAPE-MaP software calculates base flexibility (proclivity for interaction with other nucleic acids or proteins) at the individual base level, RNA SHAPE-treated inside of cells can be compared with RNA SHAPE-treated samples after extraction and deproteinization to determine where there are differences in flexibility. The locations that exhibit base flexibility clue investigators into areas that warrant further evaluation to determine (1) if there is in fact some kind of interaction occurring between the RNA of interest and some other molecule(s), and (2) what those other molecules are if there is an interaction occurring [96].

SHAPE-JuMP builds upon the concept of SHAP-MaP. Its creators credit it with being better adapted at handling long range, RNA tertiary interactions that can be more prone to errors in traditional SHAPE-MaP protocols [98]. An additional benefit of the SHAPE-JuMP procedure of RNA modeling is the structural support provided to interacting structures within the transcript [98]. Essentially, TBIA freezes the interacting RNA structures in place for the purpose of reverse transcription and sequencing [98]. The immobilization of the interacting structure makes SHAPE-JuMP a particularly effective method of predicting the structures of large RNAs [98].

While quite powerful, SHAPE-MaP is not perfect. SHAPE-MaP software is unable to differentiate between multiple different isoforms of the same transcript. Different isoforms of RNAs can be predicted by several different RNA processing events which are the results of complex RNA interactions. These interactions have impacts on the RNA’s ability to perform its intended function, whether that be translation into a functional protein or a regulatory role. In addition to the inability to differentiate between structural isoforms of an RNA transcript, SHAPE-MaP requires chemical probes that can cross the plasma membrane of the cells being studied [96]. There are several SHAPE probes commercially available, but they do not all have the same inclination to penetrate cellular plasma membranes [96]. SHAPE-MaP analysis relies on effective DNA library preparations and in RNA transcripts that are highly repetitive or structurally inaccessible, these sequencing results and predicted secondary structures are not as reliable as regions that are non-repetitive or structurally accessible [96]. SHAPE-JuMP is quite novel and not all limitations have been fully characterized. The SHAPE-JuMP creators did cite a less than optimal ability to identify tertiary contacts between interacting structures as well as a reliance on amplicon sequencing as major limitations of the protocol [98].

The applications of RNA structural modeling using SHAPE techniques are not limited to RNA in living systems. NANPs themselves take on discrete structures that are key to the effective delivery of their cargo to the appropriate location. The RNA-SHAPE techniques to produce structural models of RNA can be applied after NANP production in a quality control step to ensure that the nanoparticles accurately formed the intended structure. Additionally, SHAPE structural modeling techniques can be employed to evaluate how the structures of NANPs change when the cargo is delivered. Furthermore, we can use RNA-SHAPE to determine if the delivery process makes any changes to the structure of the nucleic acid cargo that may impact its ability to perform its intended function(s). These techniques can assist investigators in evaluating the structural stability and integrity of their delivery systems as well as cargo loads to enhance the efficacy of delivery and incorporation of the functional cargo.

## 5. Discussion

Nanotechnology has been a crucial area of research over the last decade. Nanoparticles are a growing area of academic and commercial investment, and the exploration of nucleic acid nanoparticles has opened up a wide array of research and therapeutic avenues. In the clinic, NANPs offer medical providers with potential preventative, diagnostic and therapeutic applications for patient care. Of course, as is the case with all novel technologies, there is room for nucleic acid nanoparticles to improve. Problems surrounding immunogenicity, off target activity, and unintended buildup all persist, and careful experimentation will lead researchers to optimizing specific NANPs for their unique purposes.

Outside of the clinic in a basic research capacity, NANPs are of great academic value. Investigators can use NANPs to deliver nucleic acids to knock down expression of genes to clarify the purpose of the gene. Additionally, in the arena of RNA domains, we propose the use of NANPs to deliver RNA structural domains to cells that have been depleted of the same structural domains. In this planned research, one could examine the impact of RNA structural domain loss followed by complementation of the cells with the missing domain to clarify the purpose of discrete RNA structural domains. Once optimized, this system will allow us to gain a better understanding of how RNA structure influences its function.

As research with nucleic acid nanoparticles progresses, investigators will need to address the challenges and disadvantages that come with working with them. Challenges such as unintended immune neutralization, NANP accumulation, and off-target effects are most common. The most powerful tool researchers have in mitigating these challenges is careful design of the NANPs so that they are non-immunogenic prior to target site delivery, do not build up in inappropriate locations, and do not act on inappropriate tissue. This can be done through robust screening of candidate NANPs to ensure that their specificity is as narrow as possible (i.e., it has only one complementary sequence). When designing NANPs for use in the clinic, specificity is absolutely crucial for proper delivery and appropriate immune activation. Additionally, investigators must consider the stability of the NANP they wish to design. While the NANP should be stable enough to travel to the intended target tissue, once there, it must be able to deliver its cargo and then be broken down and cleared to prevent accumulation of “spent” NANPs. The structure of the NANP is a key element in its activity and special attention must be paid to how the nanostructures will react upon arrival at the target site. Advances in NANP design technology (i.e., software like NanoTiler and SELEX) will undoubtedly assist researchers in creating NANPs that have highly specific sequences to limit target possibilities and the proper three-dimensional structures to act on a specific target, then undergo degradation and clearance [19,20,99].

## Data Availability

Not Applicable.
